# Considering medical students’ perception, concerns and needs for e-exam during COVID-19: a promising approach to improve subject specific e-exams

**DOI:** 10.1080/10872981.2022.2114131

**Published:** 2022-08-21

**Authors:** Stefanie Ziehfreund, Johannes Reifenrath, Marjo Wijnen-Meijer, Julia Welzel, Fabian Sauter, Hannah Wecker, Tilo Biedermann, Alexander Zink

**Affiliations:** a Department of Dermatology and Allergy, Technical University of Munich, School of Medicine, Munich, Germany; b Institute of General Practice and Health Services Research, School of Medicine, Technical University of Munich, Munich, Germany; c School of Medicine, TUM Medical Education Center (TUM MEC), Technical University of Munich, Munich, Germany; d Department of Dermatology, University Hospital Augsburg, Augsburg, Germany; e Department of Informatics, Technical University of Munich, Munich, Germany; f Division of Dermatology and Venereology, Department of Medicine Solna, Karolinska Institute, Stockholm, Sweden

**Keywords:** e-assessment, e-exam, curriculum, medical students, e-learning

## Abstract

The COVID-19 pandemic forced a rapid shift to digital strategies including e-exams in medical schools. However, there are significant concerns, predominately from student perspectives, and further data is required to successfully establish e-assessment in the medical curricula. The objective of the study was to examine medical students’ perceptions, concerns, and needs regarding e-assessment to establish a comprehensive e-exam based on these and previous findings and to evaluate its effectiveness in terms of examinee perceptions and further needs. During the 2021 summer term, a cross-sectional study using qualitative and quantitative methods was conducted among all 1077 students at the School of Medicine, Technical University of Munich. They were asked to provide information regarding their characteristics, preferred exam format, e-assessment perception, concerns, and needs in an online questionnaire. Based on these findings, a pilot e-exam including an e-exam preparation for the students were established and subsequently evaluated among 125 pilot e-exam examinees under study consideration via an online-questionnaire. Of the 317 pre-exam participants (73.2% female), 70.3% preferred in-person exams and showed concerns about the technological framework, privacy, and examination requirements. Qualitative analysis showed that these concerns lead to additional exam stress and fear of failure. The 34 (79.4% female) participants who participated in the evaluation survey showed a significantly more positive e-exam perception. The fairness of the platform, the independence from an internet connection, the organization including the e-exam preparation, and the consideration of participant needs were discussed as particularly positive in the open-ended comments. In both surveys, participants requested uniform platforms and processes for all subjects. This study provides evidence for a positive, complementary role of student participation in a successful e-exam implementation. Furthermore, when establishing an e-exam format in the medical curricula, e-exam training, equal accessibility, availability offline, and all-round fairness should be considered.

## Introduction

The COVID-19 pandemic has presented new challenges in medical education [1]. During mandated lockdowns, many institutions of higher education have been forced to move their instruction online to continue the educational process, which in some cases required new concepts [2–6]. Examinations could also not always be conducted on-campus to comply with hygiene requirements, which especially posed a challenge for large classes with many students. Although online assessment has for years been successively established in higher education across many countries [7,8], most German medical schools continued to hold on-campus assessments. Thus, medical schools were required to abruptly reevaluate their strategies for summative assessment.

Overall, medical students encounter practical, oral, and written exams during their studies. Remote online alternatives for practical exams can be realized e.g., through the use of dummies [9]. Oral and written exams, such as communication skill and knowledge tests, can be conducted as online video meetings and digitized as e-exams on examination platforms, respectively [10–12]. To address issues with examinee identity verification and to check the use of unauthorized aids [12,13] online proctoring technologies can be used [13,14]. However, there are significant concerns, predominately from student perspectives, about personal and data privacy and false positives [15]. Alternatively, open-book exams can also be administered, which test student abilities to quickly access and evaluate relevant information. This ability as part of clinical decision making is an important aspect of modern medical practice [16]. Therefore, testing the ability to evaluate information in a limited timeframe and with access to resources increases the validity of open-book exams [17].

Nevertheless, even open-book formats cannot counter other concerns about remote e-examination identified by several recent studies on medical students (e.g., insecure internet connection, limited access to adequate devices and space, inappropriate exam questions) [11,18,19]. These concerns and related consequences, such as additional stress, can be addressed through routine use of online learning and e-exams. If routine use is not possible, an interdisciplinary academic team is essential for student-centered testing with didactically correct use of digital methods and appropriate exam scenarios for different subject areas [20,21]. Moreover, target-group specific training can increase student e-assessement competency and decrease concerns [20,21].

This requires evidence to thoroughly understand the benefits, shortcomings, and student needs of e-exams in specific areas of study [7,17].

Accordingly, the purpose of the study was to examine medical student perceptions, concerns, and needs regarding e-assessment to establish a comprehensive e-exam based on these and previous findings and to evaluate the e-exam’s effectiveness in terms of perception and further needs of examinees. These findings can help to improve subject-specific e-exams to establish e-assessment in the medical curricula.

## Materials and methods

### Study design

A cross-sectional study was conducted using qualitative and quantitative methods to explore medical students’ perceptions, concerns, and needs to established a pilot e-exam and evaluated the pilot e-exam. Questionnaires were carried out as anonymous online surveys among medical students at the Technical University of Munich (TUM). The first cut-off point was in May 2021, prior to the first remote e-exam period at the TUM School of Medicine. The second sample point was in June 2021, following the pilot e-exam. Links to the pre-exam and evaluation questionnaires were distributed via e-mail and published on social media channels of the TUM medical school. Study participation was voluntary, and all students provided electronic informed consent prior to participation. This study was conducted in accordance with the Declaration of Helsinki and was reviewed and approved by the local ethics committee of the Medical Faculty at Technical University of Munich (reference 203/21 S).

### Setting and participants

The population under consideration was clinical medical students [22] aged 18 years and older at the TUM (N = 1,077). The School of Medicine at the TUM offers medical training for students who have successfully completed their preclinical studies. Preclinical studies in medicine are organized in cooperation between TUM and another Munich university. The students had no previous experience with remote e-exams during their clinical medical studies at the TUM, as all medical exams at the TUM were on-campus paper-based exams prior to the COVID-19 pandemic. However, experience in e-assessment was possible if students had previously encountered an e-exam during their preclinical studies or studied medicine or a different subject at another university or faculty where e-exams were already established. The pre-exam questionnaire was administered to all students enrolled in the 2021 summer term regardless of their e-exam experience and their participation in the pilot e-exam (N = 1,077) to gain the broadest possible insight into student perceptions, concerns, and needs. For the evaluation, only the 125 students who took the pilot e-exam in the 2021 summer term were eligible to evaluate their experience with the e-exam format. An exclusion criterium was the inability to fill out a German questionnaire.

### Remote e-exam

The exam for the class *gerontology* (14 lecture hours/term, scheduled for the 5^th^ clinical study term) was used as pilot model for this project because of its interdisciplinarity that includes subjects like dermatology, gastroenterology, and gynecology. The exam, coordinated by the Department of Dermatology and Allergy of the TUM, was thus far conducted as an on-campus paper-based multiple-choice exam (MCQ; 30 questions, 45 minutes). The remote e-exam was administered by a team consisting of the responsible course coordinator (AZ), an informatician experienced and trained with e-assessments (FS), a didactical professional (JW), and a research assistant (SZ). The e-exam was established in compliance with all legal requirements (Bavarian Remote Testing Regulation, General Academic and Examination Regulation of the TUM, European General Data Protection Regulation (DSGVO)) as well as based on the current literature [7,8,12]. Pre-exam survey results from 317 participants were included in the e-exam (preparation) development and implementation while considering existing tools and evidence in the literature. The following elements were elaborated by AZ, FS, JW and SZ.

EvaExam and TUMexam were available as examination platforms at the TUM. For the *gerontology* exam, TUMexam was used based on the following advantages: the system digitally assists the examination process, can be accessed with the examinee’s individual link, does not require an internet connection during examination, and offers the possibilities of remote e-exam taking with a computer/tablet or paper (print the exam, write the exam on paper, digitize, and submit). Alternatively, the *gerontology* exam could be taken as an on-campus paper-based exam for those students who did not have the appropriate environment and requirements at home. The exam was conducted in an open-book format to benefit from the aforementioned possible advantages [16,17] and address examinee concerns about proctoring. All three formats had identical presentations and content (25 MCQ and 2 very short answer questions (VSAQ) [23,24]) as well as identical test versions. The exam was scheduled for 60 minutes. In the following 5 minutes, students using digital formats were asked to begin submission. Once the start of submission was registered, there was an additional 55 minutes for the full upload to allow for network issues. During the remote e-exam, examinees could ask for assistance via chat from AZ and FS. Additionally, examinees had the option to submit periodically for security.

**Figure 1.**
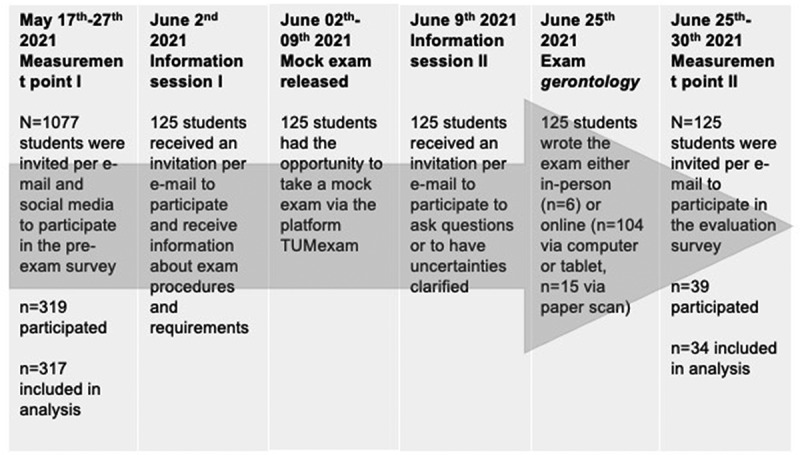
Study timeline and process.

Prior to the *gerontology* e-exam, two training sessions guided by FS, a mock remote e-exam, a user manual for TUMexam, and informational e-mails were offered to the examinees (Figure 1). The training session consisted of a presentation of the different ways of taking the exam, an introduction to TUMexam, explanation of the necessary software and hardware, and the clarification of misunderstandings (e.g., the need for a permanently stable Internet connection). The format of the mock e-exam was analogous to the later exam, using questions from prior exams supplemented by two open-ended questions. A time limit was not set so that students had sufficient time to familiarize themselves with the platform.

### Data collection

A self-administered online pre-exam and evaluation questionnaire was developed by a team of medical and educational researchers (AZ, SZ, WMM) and representatives of the student body of the TUM medical school (JR) based on the literature [12,25]. The pre-exam questionnaire was primarily used to establish the remote e-exam (preparation) and secondarily to compare results with the evaluation results. It contained no identifying information. Both questionnaires were amended based on feedback. The pilot-tests were carried out by 20 medical students from another German medical school to give all students at the TUM the possibility to participate in the survey without previously knowing the questionnaires (supplementary material 1 and 2).

### Questionnaire

The pre-exam questionnaire collected *students’ characteristics* including age (18–20, 21–23, 24–26, 27–30, and >30 years), gender (female, male, other), and clinical study term (1^st^-6^th^ term). Information about *students’ experience with e-assessment* was obtained by asking about prior participation in any e-mock exams (yes, no) and e-exams for university-level classes (0, 1–2, 3–4, >4 exams). We asked about *participation in the upcoming gerontology e-exam* (yes, no). Participants who reported no participation were asked to answer further questions on their overall perception of e-exams. We assessed the *preferred exam format* (remote, on-campus), their *reason for choice* with ‘Please justify the choice for the [exam format]’, *concerns* (‘What concerns do you have about the upcoming e-exam(s)?’), and *needs* (“What are your wishes for the upcoming e-exam?“) regarding the e-exam using free-text comments. Participants also completed a modified version of the *Student Perceptions of e-Assessement Questionnaire* (*SPEAQ*), a questionnaire developed by Dermo to identify possible risks in planning e-assessment and to assess student opinion [25]. The *SPEAQ* was developed by Dermo using an adapted Likert scale to convert attitudes and feelings regarding e-assessment into numbers to draw conclusions and assist effective decision making [25]. This is especially important in the area of education during the COVID-19 pandemic. A considerable time was spent at the outset of development of the *SPEAQ* by Dermo to test its validity. The English language questionnaire was initially delivered to 130 undergraduates of different subjects who had taken part in an e-exam (formative or summative) during the 2007–2008 academic year, showing poor to good internal consistency reliability (α = .331-.826) [25]. In this study *SPEAQ* was translated into the German language and adapted in accordance with the pre-exam period following the OECD’s current position paper on e-exams [12] and previous literature [7,8,26,27] without modifying the existing dimensions and scales. An item universe was generated by our research team and assessed in terms of validity. The dimension indicators were finalized after pre-test feedback. For each of the 30 indicators in the 6 dimensions (affective factors, validity, practicality, reliability, security, and teaching and learning; [Table 1]), participants could respond on a 5-point Likert scale from 1 (‘strongly agree’) to 5 (‘strongly disagree’). Dimensions showed poor to good internal consistency reliability in this sample with Cronbach’s alphas of .502 to .864 (Table 1).

**Table 1.** Overall scores of the 6 dimensions of the SPEAQ for pre-exam (n = 317) and post-exam (n = 34) questionnaires.

Dimension	N of items		Median (IQR)*		Test-statistic U P**	Cronbach’s alpha***	
	*pre-exam*	*evaluation*	*pre-exam*	*evaluation*	-	*pre-exam*	*evaluation*
Affective factors	7	7	2.29 (1.71–3.14)	2.92 (1.96–3.64)	4,127.50.025	.864	.849
Validity	5	5	3.20 (2.60–3.60)	3.40 (2.80–4.00)	4,078.00.019	.502	.497
Practicality	6	6	2.83 (2.33–3.50)	3.79 (3.33–4.21)	2,249.50<.001	.757	.612
Reliability	3	3	2.00 (1.33–2.67)	3.00 (2.33–4.00)	2,386.50<.001	.520	.597
Security	5	5	3.00 (2.60–3.69)	3.60 (3.15–4.00)	3,243.50<.001	.692	.437
Learning and teaching	4	3****	2.50 (1.75–3.25)	3.00 (3.00–3.75)	4,530.00.126	.801	.792

* Ranging from 1.0 (negative rated) – 5.0 (positive rated) with a neutral mid-point of 3.0 (3.00 was considered neutral, <3.00 negative, and >3.00 positive).

** p-value was calculated using Mann-Whitney U test, α = .05*** Internal consistency of the six dimensions has been measured with Cronbach’s coefficient alpha

**** Because the exam was conducted digitally and in-person, the item ‘E-exams provide opportunities for knowledge testing that would not be possible with in-person examinations’ was removed from the evaluation survey.

*IQR, interquartile range*

The evaluation questionnaire contained the same questions about *students’ characteristics* as the pre-exam questionnaire. Subsequently, students were asked about the usage of *preparation materials* (mock exam via TUMexam or other platforms, information sessions, the manual ‘e-exams with TUMexam’) and their efficacy. Using free-text questions, students were asked to explain their choice of *exam format* (remote using a computer/tablet, remote using paper, on-campus): ‘Please justify the choice for the [exam format].’ They were then prompted to answer the question ‘If given the choice, would you choose the [exam format] again?’ (yes, no), with the option of providing further explanation (‘Please justify the choice’). The items from the adapted version of the *SPEAQ* were modified to evaluate the *gerontology* exam according to the same process as the pre-exam questionnaire. Dimensions showed poor to good internal consistency reliability in this sample with Cronbach’s alphas of .437 to .849 (Table 1).

### Statistical analyses

All data were coded prior to data analysis. Only participants who answered 100% of items validly were considered. Tests of normality were performed where appropriate using normal probability plots and Shapiro-Wilk test. Descriptive data were analyzed using absolute and relative frequencies for categorical variables and median with interquartile range (IQR) for quantitative variables. For analysis of the *SPEAQ*, we created overall ratings for each of the 6 dimensions by combining the indicator ratings, resulting in one 13-, 17-, 25-, 29- and two 21-point rating scale ranging from 1.0–5.0 with a neutral mid-point of 3.0. Positively recorded indicators had been recoded so that ratings could be combined. Further, we considered the 30 individual indicators of the *SPEAQ*. Median ratings of 3 were considered neutral, <3 negative, and >3 positive. Same analyses were performed for the evaluation dataset. Differences between pre-exam and evaluation *SPEAQ* data were analyzed using Mann-Whitney U tests. For all analyses, the level of significance was set as α = .05. Internal consistency reliability was assessed by Cronbach alpha (α).

Free-text answers were analyzed using the qualitative content analysis according to Mayring [28]. Categories were developed deductively from the guided questions and supplemented by categories that resulted from the material by SZ. Another researcher (author AZ) examined these proposed categories for traceability. Unclear passages were discussed and, if necessary, changed and adapted by consensus (SZ, AZ). This resulted in a category tree with defined anchor examples and the subsequent definition of subcategories (SZ). The categories were discussed and proved to be comprehensive (SZ, AZ). All materials were coded according to these categories (SZ) and discussed in case of ambiguity in coding (SZ, AZ). Additionally, qualitative results were quantified for an overview of the frequency of the emerged topics.

Quantitative analyses were conducted using IBM SPSS Statistics (Version 26, IBM Corporation, Armonk, NY, USA). MAXQDA software (version 12) was used for coding and identifying categories.

## Results

### Pre-exam

In total, 320 students participated in the pre-exam survey. Three participants with insufficient data were excluded. Of the remaining 317 participants (73.2% female; response rate = 29.4%), 94 stated to choose the remote e-exam, with higher acceptance among participants with prior e-exam experience than those without (43.6% and 29.1%, respectively). Most participants, however, had no prior experience with mock e-exams or e-exams (68.1% and 66.6%, respectively; Table 2).

**Table 2.** Characteristics of participants of pre-exam and evaluation questionnaires.

	Pre-exam [n (%)]	Evaluation [n (%)]
Total	317 (100)	34 (100)
Female	232 (73.2)	27 (79.4)
Male	83 (26.2)	7 (20.6)
Other	2 (0.6)	0 (0)
18–20 years old	6 (1.9)	0 (0)
21–23 years old	174 (55.9)	14 (41.2)
24–26 years old	91 (28.7)	16 (47.1)
27–30 years old	23 (7.3)	3 (8.8)
> 30 years old	23 (7.3)	1 (2.9)
Clinical semester		
1^st^	21 (6.6)	0 (0)
2^nd^	61 (19.2)	1 (2.9)
3^rd^	21 (6.6)	3 (8.8)
4^th^	78 (24.6)	3 (8.8)
5^th^	50 (15.8)	22 (64.7)
6^th^	86 (27.1)	5 (14.7)
Exam format		
E-exam*	94 (29.7)	NA
In-person paper-based**	223 (70.3)	
Mock (TUM)exam*** participation		
Yes	101 (31.9)	31 (91.2)
No	216 (68.1)	3 (8.8)
Information session participation	NA	
Yes		12 (35.3)
No		22 (64.7)
Manual ‘e-exams with TUMexam’	NA	
Yes		19 (55.9)
No		15 (44.1)
E-exam experience		
0	211 (66.6)	0 (0)
1–2	88 (27.8)	3 (8.8)
3-4	12 (3.8)	8 (23.5)
>4	6 (1.9)	23 (67.6)
Exam gerontology participation****		
Yes	94 (29.7)	34 (100)
No	223 (70.3)	*NA*

NA, not applicable * E-exam is conducted off campus without supervision and with several digital submission options. ** In-person paper-based exam is held at the Technical University Munich with in-person proctoring. *** Pre-exam questionnaire asked about participation in any mock e-exam, while evaluation questionnaire specifically asked about participation in the mock e-exam on TUMexam. **** Participation in the exam for the cross-disciplinary subject gerontology in the summer term 2021.

Ratings of the six *SPEAQ* dimensions were almost neutral for ‘validity’ (median = 3.20; IQR = 1.00), ‘security’ (median = 3.00; IQR = 1.00), and ‘practicality’ (median = 2.83; IQR = 1.17). Ratings were negative for ‘learning and teaching’ (median = 2.50; IQR = 1.50), ‘affective factors’ (median = 2.29; IQR = 1.43), and ‘reliability’ (median = 2.00; IQR = 1.33) (Table 1).

Four of the thirty *SPEAQ*-indicators were rated positive, eleven neutral, and fifteen negative (Table 3). Technical malfunctions of the exam platform were a major concern (median = 1.00; IQR = 0.00; 95.9% agreed) followed by poor internet connections (median = 1.00; IQR = 1.00; 88.3% agreed) and the additional burden of different class subjects hosting e-exams on different platforms (median = 1.98; SD = 1.17; 72.6% agreed [Table 3]). Participants agreed the most that online exams use less paper (73.2% agreed). All participants reported having the necessary equipment and environment for an e-exam.

**Table 3.** Overall ratings of the 30 indicators of the *SPEAQ* for pre-exam (n = 317) and evaluation (n = 34) questionnaires.

Dimension/Item	Median (IQR)*		Test-statistic U	P-value****
**Affective factors**	**Pre-exam**	**Evaluation**	-	-
E-exams** are more stressful for me than in-person exams***.	2.00 (1.00–3.00)	2.00 (1.00–4.25)	5,017.00	.494
I prefer to write in-person exams rather than e-exams.	1.00 (1.00–3.00)	3.00 (1.00–5.00)	3,205.50	**<.001**
E-exams and in-person exams trigger the same level of stress in me.	2.00 (2.00–4.00)	2.50 (2.00–3.25)	5,129.00	.635
I think e-exams should play a more important role at the School of Medicine.	2.00 (1.00–4.00)	3.00 (1.00–5.00)	4,245.00	.**037**
In e-exams I have difficulties concentrating on the questions.	3.00 (2.00–4.00)	3.00 (2.00–4.25)	5,063.00	.554
I prefer e-exams because I am used to working on the computer.	2.00 (1.00–3.00)	2.50 (1.75–4.00)	3,638.50	.**001**
With e-exams, I miss the social interaction with my fellow students.	2.00 (1.00–4.00)	2.50 (1.00–3.75)	5,150.00	.658
**Validity**				
An e-exam is a suitable exam format for the lecture series *gerontology*.	3.00 (3.00–4.00)	4.00 (2.00–5.00)	4,272.50	.**041**
E-exams test not only my knowledge but also my IT skills.	4.00 (2.00–5.00)	3.00 (1.00–3.25)	3,550.50	**<.001**
E-exams are a contemporary form of examination.	4.00 (3.00–4.00)	4.00 (3.00–5.00)	4,371.00	.061
A too tight time limit is set for e-exams.	3.00 (2.00–3.00)	4.00 (2.00–5.00)	3,679.50	.**002**
The requirement level of the exam questions is much higher with the e-exam.	2.00 (2.00–3.00)	3.00 (2.00–5.00)	3,735.00	.**002**
**Practicality**				
I think it’s positive that online exams use less paper.	4.00 (4.00–5.00)	5.00 (4.00–5.00)	4,532.00	.099
I am afraid that my performance in the e-exam will be affected by a poor internet connection.	1.00 (1.00–2.00)	5.00 (4.00–5.00)	505.50	**<.001**
I do not have the necessary equipment to write an e-exam.	3.00 (3.00–5.00)	5.00 (4.00–5.00)	4,002.50	.**009**
I do not have an appropriate environment off campus to write an e-exam.	3.00 (2.00–5.00)	5.00 (4.00–5.00)	3,877.00	.**005**
E-exams are easier for me to access than in-person exams.	2.00 (1.00–3.00)	3.00 (2.00–5.00)	3,792.00	.**003**
When different subjects host e-exams on different platforms, it adds to my burden as a student.	2.00 (1.00–3.00)	2.00 (1.00–3.00)	5,078.50	.556
**Reliability**				
Technical malfunctions of the exam platform may affect the exam procedure.	1.00 (1.00–1.00)	5.00 (3.00–5.00)	643.50	**<.001**
Digital MC questions are more reliable than in-person exams because transcription errors are avoided.	3.00 (1.00–4.00)	3.00 (2.00–4.00)	5,225.00	.764
In-person exams are fairer than e-exams.	2.00 (1.00–3.00)	2.50 (1.00–3.25)	5,211.50	.774
**Security**				
E-exams are just as secure as in-person exams.	3.00 (2.00–3.50)	3.00 (2.00–4.00)	4,334.00	.054
I have confidence in the data security of the audit software.	4.00 (3.00–5.00)	5.00 (4.00–5.00)	4,067.00	.**014**
It is easier to cheat in e-exams than in in-person exams.	2.00 (1.00–3.00)	2.00 (1.75–3.00)	5,039.50	.519
Hacker attacks are an acute and serious problem for TUMexam.	3.00 (3.00–4.00)	4.00 (3.00–5.00)	3,515.00	**<.001**
Username and password provide a sufficient level of security for e-exams.	3.00 (3.00–4.00)	5.00 (3.00–5.00)	3,438.00	**<.001**
**Learning and teaching**				
E-exams provide opportunities for knowledge testing that would not be possible in in-person examinations.	2.00 (1.00–3.00)	NA*****	-	-
E-exams could support my learning process.	2.00 (1.00–3.00)	3.00 (1.00–4.00)	4,512.00	.108
E-exams are just a gimmick that do not help me learn.	3.00 (2.00–4.00)	3.50 (1.75–5.00)	4,552.50	.128
E-exams go hand in hand with online learning.	3.00 (2.00–4.00)	3.00 (1.00–4.00)	5,347.00	.940

* Ranging from 1.0 (negative rated) – 5.0 (positive rated) with a neutral mid-point of 3.0 (3.00 was considered neutral, <3.00 negative, and >3.00 positive).

** E-exam is conducted off campus without supervision and with several digital submission options.

*** In-person paper-based exam is held at the Technical University Munich with in-person proctoring.

**** P-value was calculated using Mann-Whitney U test, α = .05

***** Because the exam was carried out digitally and in-person, the item ‘E-exams provide opportunities for knowledge testing that would not be possible with in-person examinations.’ was removed from the post-exam survey.

IQR, interquartile rage

### Qualitative data

In total, 297/317 participants provided reasons for their choice of exam format and explained their concerns and needs regarding e-exams. Overall, 936 text segments were grouped into five categories. The technical framework (first category) was the most frequently stated concern (k = 499), which referred to participants’ technical concerns (k = 221) and fears regarding internet connectivity (k = 278). The second category of ‘overall exam framework’ (k = 301) addressed concerns about the exam environment and was further grouped in ‘lack of adequate exam atmosphere’ (k = 199) and ‘lack of usual exam condition’ (k = 102). The third category of ‘examination requirements’ (k = 108) were divided in the demand for equality of the exam (k = 72) and their fear of the unknown exam format (k = 36). Finally, the students discussed privacy concerns (category 4, k = 9) and the ‘lack of social interaction’ (category 5, k = 19; [Table 4]). Time pressure, additional exam stress, and fear of exam failure were frequently discussed as consequences of categories 1–4.

**Table 4.** Overview of the categories and subcategories of the open-ended questions in pre-exam and evaluation surveys. Pre-exam statements (n = 297, k = 936) were considered in the design of the remote e-exam (preparation) to assuage the concerns and fears of examinees. Evaluation statements (n = 34, k = 145).

	Category Subcategory	k*	Example
Pre-exam	Technical framework	499	-
	technical concerns	221	*‘[…] fear of technical failure and to fail the exam as a consequence.’*
	fears regarding internet connectivity	278	*‘I feel uncomfortable when the functioning of my exam depends on my […] internet connection. This adds additional stress.’*
	Overall exam framework	301	-
	lack of adequate exam atmosphere	199	*‘stronger “exam feeling”, i.e., somehow more stress, so that I work more concentrated like at home.’*
	lack of usual exam condition	102	*‘If you have any questions, you can ask the instructor right away if the exam is on-campus.’*
	Examination requirements	108	-
	unknown exam format	36	*‘[…] the exam will take place in new unknown format, which causes uncertainty.’*
	exam equivalence	72	*‘The new system will be relaunched with questions that are tougher and will fail us.’*
	Privacy concerns	9	*‘Privacy issues with monitored exams, […] almost an invasion of privacy.’*
	Lack of social integration	19	*‘It’s nice to cross paths with fellow students at least during the exams if you don’t do that during the semester already.’*
Evaluation	Advantages	83	-
	fairness of the online platform	18	*‘In terms of platform, I found TUMExam super and VERY much fairer than EvaExam (other exam platform used), also with the unlimited access.’* *‘The option of being able to submit PDFs periodically for security purposes is very commendable.’*
	independency of internet connection	20	*‘A permanent Internet connection is no longer required (see TUMexam), because the worry of losing the Internet connection was still the most stressful.’*
	overall organization	19	*‘The preparation could not have been better.’* *‘I also found it very good that for the gerontology exam there was an alternative offer to write the exam in paper format at the university.’* *‘I found that it was excellent, online exam for president’*
	inclusion of students needs	15	*‘I liked that our needs were taken into consideration when designing the exam. It has also contributed to the motivation to take the e-exam.’*
	exam equivalence	11	*‘Questions were fair and not harder than in prior exams. Moreover, I think it was great to have some short answer questions.’*
	Disadvantages	22	-
	writing interface (Macintosh operating system)	2	*‘[…] difficulties with free text tasks. These were not saved (editing on Mac)’*
	limited user experience	20	*‘[…] elaborate processing, normal system of EvaExam (other system used) better to use.’*
	Further needs	25	-
	e-exam training	9	*‘Video recording of the information session regarding TUMExam would have been nice.’*
	uniform exam platform	8	*‘A uniform platform, […] unnecessary stress as a result and really avoidable.’*
	further e-exams	8	*‘I would appreciate further e-exams.’*
	Change of mind regarding e-exams	15	*‘I would choose the online exam again. Because it worked out great – against other fears.’* *‘Independence from the internet connection was great and took the initial concerns away. Accordingly, I would use this format again in the future.’*

* test segments that were categorized

### Evaluation and comparison of both surveys

Thirty-nine students participated. Five participants with insufficient data were excluded. The remaining 34 participants (79.4% female; response rate = 27.2%) passed the remote e-exam (97.1% computer-based, 2.9% paper-based). Twenty-nine participants (85.3%) indicated that they would choose the remote e-exam again. Overall, 33 participants stated having used at least one of the *gerontology*-specific preparation measures, with 31 using the TUMexam mock exam, 19 using the manual, and 12 visiting the information session (Table 2). The majority rated the information session (83.3%) and the TUMexam mock e-exam (80.6%) as useful, while the manual was considered useful by 42.1% of participants (Table 2).

Ratings of the six *SPEAQ* dimensions were positive for ‘practicality’ (median = 3.75; IQR = 0.88), ‘security’ (median = 3.60; IQR = 0.85), and ‘validity’ (median = 3.40; IQR = 1.20), and almost neutral for ‘reliability’ (median = 3.00; IQR = 1.67), ‘learning and teaching’ (mean = 3.00; IQR = 1.83), and ‘affective factor’ (median = 2.92; IQR = 1.86). Ratings in 5 dimensions were significantly higher compared to pre-exam values (p < .001-.025; [Table 1]). Only ‘teaching and learning’ showed no significant changes (p = 0.126).

Eleven *SPEAQ*-indicators were rated positive, nine neutral, and eight negative. One of the most negatively rated items was that of using different exam platforms (median = 2.00; IQR = 2.00) with 24 agreements (70.6%) followed by the absence of social interaction (median = 2.00; IQR = 2.00; 61.8% agreed) and the ease of cheating during e-exams (median = 2.00; IQR = 2.00; 55.9% agreed). Positive ratings were given to paper saving, internet connection experience, TUMexam’s technical features, and data security. All participants reported having the necessary equipment and environment to write an e-exam. Regarding the median, ratings in 18 indicators were higher compared to pre-exam values, with 14 indicators showing significant changes (p < .001-.045, [Table 3]), while nine indicators showed no differences in the median. Significantly less examinees agreed with the indicator that e-exams also test IT-skills (p < .001).

### Qualitative data

Overall, 145 text segments (n = 34) were classified into four categories. The most discussed category emerged was ‘advantages’ of the e-exam (k = 83), including the subcategories ‘fairness of the online platform’ (k = 18), the ‘independency of internet connection’ (k = 20), the ‘overall organization’ (k = 19), the inclusion of the students’ needs (k = 15), and the ‘equivalence of the exam’ (k = 11). In contrast, the second category referred to experienced disadvantages (k = 22) including limited user experience (k = 20) and two issues with the user interface among Macintosh operating system users. Additionally, ‘further needs’ (k = 25) including the subcategories ‘e-exam training’ (k = 9; e.g., video recording of the information session), ‘uniform platforms’ (k = 8), and the wish for ‘further e-exams’ emerged as relevant aspects from students’ point of view. Statements in the last category ‘changes of mind regarding e-exam’ (k = 15) often related to reported e-exam advantages, e.g., *‘Independence from the internet connection was great and removed the initial concerns. Accordingly, I would use this format again in the future.’* This category allowed conclusions to be drawn about changes in student perception regarding e-assessment.

## Discussion

This study aimed to understand medical students’ perceptions, concerns, and needs regarding e-exams to optimize their first clinical medical remote e-exam and to evaluate their experience with the following e-assessment. This information can help optimize digital examinations and establish a viable digital format in the medical education curricula with equitable opportunities for all students.

The pre-exam results revealed clear concerns and negative perceptions regarding security (e.g., data protection), validity (e.g., appropriateness of the online exam format), and especially technical failure. Students who participated in the e-exam evaluation survey showed a more positive perception and reported changes in their opinions of e-exams. Most participants rated e-exam positively and appreciated that their needs were considered in the design of the exam. In both surveys, the participants valued the uniform test organization (i.e., same exam platforms, clear instructions) and fair and equal examination conditions (e.g., same level of difficulty, on-campus alternative).

Medical students’ perceptions and concerns regarding e-exams identified in our pre-exam survey are in line with earlier findings and include, e.g., technical and privacy concerns or inappropriate exam questions [8,11,18–20]. However, the six dimensions of the *SPEAQ* were more negatively rated in the present study compared with the results of Dermo, who delivered the questionnaire to undergraduates at the University of Bradford [25]. While Dermo showed neutral to positive ratings, dimensions were rated negative to neutral in our study [25]. The lower ratings may be due to the limited e-exam experience, as students from the University of Bradford studied other subjects, namely management, informatics, and engineering, which reflect the main areas of use of e-assessment [25]. This is understandable, as students with experience are more adapted to the use of online systems [29,30]. Although some of the students in this study had previous experience with e-exams, this was not part of their clinical studies at the TUM and most importantly, not part of routine practice in learning and assessment. However, routine in e-learning and e-assessment is necessary to boost self-confidence and enhance learning outcomes.

Furthermore, 70% of our pre-exam participants were unwilling to write an e-exam, which is higher than expected based on the literature [11,31,32]. The fact that the e-exam took place during a global pandemic should be considered. Undoubtedly lockdowns had unpredictable effects on student social life, learning, revision, and mental well-being as students adapted to a new normal [33]. Coupled with the rapid shift to e-lectures and e-exams, this may have contributed to the increased reluctance of medical students to take e-exams to maintain normality at least within this framework. A previous study reported that students’ perceived efforts and time needed to prepare for e-exams was higher compared to on-campus exams. This was found to be associated with an inclination towards on-campus exams [32]. The time and effort required could be related to the variety of teaching methods and different study materials/resources used during distance learning and may be another explanation for the high reluctance in this study.

These observations made it important to consider student needs identified in the pre-exam survey during the conception of the *gerontology* e-exam not only to eliminate unnecessary concerns (e.g., the need for a permanently stable Internet connection, privacy concerns, technical uncertainties), but also to integrate them into the e-exam development process. Recent studies have reported that student participation has demonstrated positive effects on the curriculum development process and student learning motivation and success [34,35]. The same could also be applied to the conception of examinations. Our data provides support for this, as analysis of qualitative evaluation data found that the participants appreciated that their needs were considered. The combination of gaining e-exam experience [20,31] and feeling involved in the development process may explain why students showed more positive perceptions after the exam. Finally, this is reflected in the high participation rate for the digital exam format (5% in-person, 95% e-exam) and the large proportion of participants who are interested in further e-exams.

Interestingly, the advantages in teaching and learning due to e-examination and the consideration of IT-skills in an open-book e-exam were rated slightly higher and lower after the e-exam, respectively. To realize the potential of the e-exam by assessing the ability of students to quickly access and evaluate relevant information, [16,17] these aspects, disadvantages (e.g., limited user-experience), and further needs (e.g., video recorded e-exam training) identified in this study should be considered in planning of e-assessments.

In the context of medical education, future research should investigate challenges in improving e-learning infrastructure [36] and possibilities for continuous self-assessments or virtual reality-based e-assessment applications [9,37], ideally through cooperation between interdisciplinary academic teams and students.

Limitations of our study include the relatively low response rate of 29% at the pre-exam survey and 27% at the evaluation survey, the non-participation of the 6 in-person examinees, and a participant gender ratio that is not representative of medical students at the TUM. Additionally, we cannot exclude that mostly students with a particularly high level of interest participated. This may contribute to an underestimation of the number of concerns. Contrary to this, an above-average participation of students who wanted to share their concerns and fears about the e-exam may have resulted in an overestimation of the number of concerns. However, this can be beneficial in addressing the study’s aim to identify a broad range of concerns and needs. A direct pre-post comparison was not performed in our study. Nevertheless, this is to our knowledge the first study to identify medical student perceptions of e-exams prior to testing to adjust the e-examination process followed by a subsequent evaluation. Moreover, the use of open-ended questions at the beginning of the study questionnaire permitted students to provide honest feedback unaffected by *SPEAQ* rating scales. We did not use a validated questionnaire to achieve the most effective data for the purpose of the study. Additionally, it should be considered that the modified SPEAQ showed barely adequate or inadequate levels of consistency for 3 of 6 dimensions. However, measures were taken to create a reliable and practical research tool according to guides for developing questionnaires [26,27].

## Conclusion

Our findings indicate that despite initial concerns, remote e-exams can be established as an alternative format to traditional assessments in medical education. During implementation, student needs and subject-specific conditions should be considered. In this context, target-specific e-exam training can be advantageous. The inclusion of student participation and academic teams consisting of technical and didactic experts in e-exam conception can reduce student fears and additional exam stress.

## Supplementary Material

Supplemental Material

## Data Availability

The data that support the findings of this study are available from the corresponding author, SZ, upon reasonable request.
