# Supplementary material for: Considering medical students’ perception, concerns and needs for e-exam during COVID-19: a promising approach to improve subject specific e-exams
Source: Med Educ Online. 2022 Aug 21;27(1):2114131. doi: 10.1080/10872981.2022.2114131 (PMC9397442; doi:10.1080/10872981.2022.2114131)
Supplement: Supplemental Material [file ZMEO_A_2114131_SM1227.docx]

Supplementary material 1
Final pre-exam questionnaire in original language (German)

**Wird in diesem Fragebogen von Online-Klausuren gesprochen, handelt es sich immer um das**

**Format einer Fernklausur die Sie außerhalb des Campus ohne menschliche Aufsicht erledigen können.**

Geschlecht

weiblich


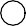

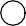

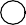


männlich

diverse

Alter

18-20 Jahre


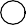

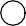

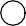

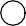

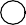


21-23 Jahre

24-26 Jahre

27-30 Jahre

> 30 Jahre

In welchem klinischen Semester studieren Sie derzeit?


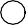
 1
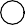
 2
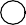
 3
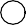
 4
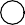
 5
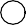
 6

Haben Sie bereits an einer Online-Probeklausur teilgenommen?


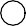
 Ja
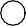
 Nein

Bitte kennzeichnen Sie, wieviele Online-Klausuren Sie bereits an einer Hochschule oder Universität abgelegt haben:

0


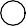

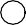

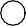

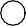


1-2

3-4

> 4

Sind Sie zur Klausur zur Vorlesung Medizin des Alterns und des alten Menschen (MdA) im Sommersemester 2021 angemeldet?


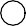
 Ja
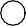
 Nein

Sofern Sie nicht an der Klausur zur Vorlesung Medizin des Alterns und des alten Menschen (MdA), die im SS 21 zum ersten Mal als Online-Klausur stattfinden wird, teilnehmen werden, bitten wir Sie, die nachfolgenden Fragen/Aussagen auf Basis Ihrer Erfahrung mit Online-Klausuren oder Erwartungen hinsichtlich Online-Klausuren im Fach Medizin zu beantworten/bewerten.

Welches Prüfungsformat haben Sie für die bevorstehende Klausur zur Vorlesung MdA gewählt (würden Sie wählen)?

Online-Klausur


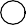

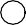


Papier-basierte Prüfung am Campus

Sie haben sich für die Online-Klausur/ Papier-basierte Prüfung am Campus entschieden. Bitte begründen Sie Ihre Wahl:

______________________________________________________________________________________________________________________________________________________

Welche Bedenken haben Sie hinsichtlich (der bevorstehenden) Online-Klausur(en)?

______________________________________________________________________________________________________________________________________________________

Welche Wünsche haben Sie in Bezug auf (die bevorstehende) Online-Klausur(en)?

______________________________________________________________________________________________________________________________________________________

**Inwiefern stimmen Sie den folgenden Aussagen zu?**

|  | Ich stimme voll zu |  |  |  | Ich stimme gar nicht zu |
| --- | --- | --- | --- | --- | --- |
| Online-Klausuren sind für mich stressiger als Papier-basierte Prüfungen. |  |  |  |  |  |
| Ich schreibe lieber  Papier-basierte Prüfungen als Online-Klausuren. |  |  |  |  |  |
| Online-Klausuren und  Papier-basierte Prüfungen lösen in mir ein gleich hohes Stressniveau aus. |  |  |  |  |  |
| Ich denke Online-Klausuren sollten eine wichtigere Rolle an der Fakultät für Medizin spielen. |  |  |  |  |  |
| In Online-Klausuren habe ich Schwierigkeiten mich auf die Fragen zu konzentrieren. |  |  |  |  |  |
| Ich bevorzuge Online-Klausuren, weil ich es gewohnt bin, am PC zu arbeiten. |  |  |  |  |  |
| Bei Online-Klausuren fehlt mir die soziale Interaktion mit meinen KommilitonInnen. |  |  |  |  |  |

**Inwiefern stimmen Sie den folgenden Aussagen zu?**

|  | Ich stimme voll zu |  |  |  | Ich stimme gar nicht zu |
| --- | --- | --- | --- | --- | --- |
| Eine Online-Klausur ist ein geeignetes Prüfungsformat für die Vorlesungsreihe Medizin des Alterns (für die Vorlesungsreihen im Fach Medizin). |  |  |  |  |  |
| Online-Klausuren testen nicht nur mein Fachwissen, sondern auch meine IT- Kompetenz. |  |  |  |  |  |
| Online-Klausuren sind eine zeitgemäße Prüfungsform. |  |  |  |  |  |
| Für Online-Klausuren wird ein zu knappes Zeitlimit angesetzte. |  |  |  |  |  |
| Das Anforderungsniveau der Klausurfragen wird durch die Alternative der Online-Klausur unangebracht höher als in herkömmlichen Papierbasierten Prüfungen. |  |  |  |  |  |

**Inwiefern stimmen Sie den folgenden Aussagen zu?**

|  | Ich stimme voll zu |  |  |  | Ich stimme gar nicht zu |
| --- | --- | --- | --- | --- | --- |
| Ich bewerte es positiv, dass die Online-Klausur weniger Papier verbraucht. |  |  |  |  |  |
| Ich befürchte, dass mein Abschneiden in der  Online-Klausur durch eine schlechte Internetverbindung beeinträchtigt wird. |  |  |  |  |  |
| Ich verfüge nicht über die notwendige Ausstattung eine Online-Klausur zu schreiben. |  |  |  |  |  |
| Ich habe außerhalb des Campus keine angebrachte Umgebung, um eine Online-Klausur zu schreiben. |  |  |  |  |  |
| Online-Klausuren sind für mich leichter zugänglich als Papier-basierte Prüfungen. |  |  |  |  |  |
| Wenn verschiedene Fächer unterschiedliche Plattformen  (name oft he platform, EvaExam) zur Ausrichtung der Online-Klausur nutzten, trägt dies zu meiner Belastung als Studierender bei. |  |  |  |  |  |

**Inwiefern stimmen Sie den folgenden Aussagen zu?**

|  | Ich stimme voll zu |  |  |  | Ich stimme gar nicht zu |
| --- | --- | --- | --- | --- | --- |
| Technische Störungen der Prüfungsplattform können den Klausurablauf beeinträchtigt. |  |  |  |  |  |
| Die Online-MC-Fragen sind zuverlässiger als Papier-basierte Prüfungen, da Übertragungsfehler vermieden werden. |  |  |  |  |  |
| Papier-basierte Prüfungen sind fairer als die Online-Klausur. |  |  |  |  |  |

**Inwiefern stimmen Sie den folgenden Aussagen zu?**

|  | Ich stimme voll zu |  |  |  | Ich stimme gar nicht zu |
| --- | --- | --- | --- | --- | --- |
| Online-Klausuren sind genauso sicher, wie die Papier-basierte Prüfungen. |  |  |  |  |  |
| Ich habe Vertrauen in die Datensicherheit der  Prüfungssoftware (name of the platform). |  |  |  |  |  |
| Es ist in Online-Klausuren leichter zu betrügen als in  Papier-basierten Prüfungen. |  |  |  |  |  |
| Hacker-Angriffe stellen ein akutes und ernstzunehmendes  Problem für (name oft he paltform) dar. |  |  |  |  |  |
| Personalisierter Link und Matrikelnummer bieten ein  ausreichendes Sicherheitsmaß für die Online-Klausuren. |  |  |  |  |  |

**Inwiefern stimmen Sie den folgenden Aussagen zu?**

|  | Ich stimme voll zu |  |  |  | Ich stimme gar nicht zu |
| --- | --- | --- | --- | --- | --- |
| Online-Klausuren verfügen über Möglichkeiten der Wissensabfrage, die in Papierbasierten Prüfungen nicht möglich wären. |  |  |  |  |  |
| Online-Klausuren könnten meinen Lernprozess unterstützen. |  |  |  |  |  |
| Die Online-Klausur sind nur eine Spielerei, die mich nicht beim Lernen unterstützen. |  |  |  |  |  |
| Online-Klausuren gehen Hand in Hand mit Online-Learning. |  |  |  |  |  |

Supplementary material 2
Final post-exam questionnaire in original language (German)

Geschlecht

weiblich


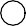

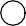

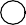


männlich

diverse

Alter

18-20 Jahre


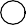

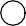

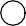

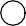

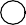


21-23 Jahre

24-26 Jahre

27-30 Jahre

> 30 Jahre

In welchem klinischen Semester studieren Sie derzeit?


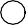
 1
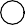
 2
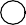
 3
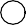
 4
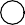
 5
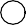
 6

**Wird in diesem Fragebogen von Online-Klausuren gesprochen, handelt es sich immer um das**

**Format einer Fernklausur die Sie außerhalb des Campus ohne menschliche Aufsicht erledigen können.**

Bitte kennzeichnen Sie, wieviele Online-Klausuren Sie bereits an einer Hochschule oder Universität abgelegt haben:

0


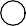

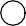

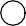

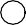


1-2

3-4

>4

**Bitte nehmen Sie bei den folgenden Fragen Bezug auf Ihre Erfahrung mit der Klausur zur**

**Vorlesung QS Medizin des Alterns, welche in diesem Semester erstmalig als digitale Klausur über die Plattform (name oft he platform) angeboten wurde.**

Welche Möglichkeit(en) haben Sie zur Vorbereitung auf die Online-Klausur genutzt? (Mehrfachantworten möglich)

Angebot der Probe-Klausur 'QS Medizin des Alterns via (name of the platform)' Angebot der Informationsveranstaltung am 02.06/09.06.

Manual 'Digital Exams with (name of the platform)'

Teilnahme an anderen Online-(Probe-)Klausur

selbständige (online) Recherche

sonstige

keine

**Inwiefern stimmen Sie den folgenden Aussagen zu?**

|  | Ich stimme voll zu |  |  |  | Ich stimme gar nicht zu |
| --- | --- | --- | --- | --- | --- |
| Die Probeklausur hat mich gut auf die Online-Klausur vorbereitet |  |  |  |  |  |
| Die Informationsveranstaltung  hat mich gut auf die Online-Klausur vorbereitet |  |  |  |  |  |
| Das Manual hat mich gut auf die Online Klausur vorbereitet |  |  |  |  |  |

Welche zusätzliche Information/Unterstützung hätten Sie sich für die Vorbereitung gewünscht?

______________________________________________________________________________________________________________________________________________________

In welcher Form haben Sie die Klausur zur Vorlesung QS Medizin des Alterns abgelegt?

Online-Klausur - digitale Bearbeitung via Computer oder


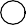

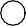

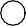


Tablet Online-Klausur - Bearbeitung auf dem Papier

Papier-basierte Prüfung am Campus

Bitte begründen Sie Ihre Wahl:

______________________________________________________________________________________________________________________________________________________

Würden Sie sich bei einer Wahlmöglichkeit erneut für eine Online-Klausur entscheiden?

Ja


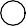

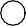


Nein

Bitte begründen Sie Ihre Entscheidung:

______________________________________________________________________________________________________________________________________________________

**Inwiefern stimmen Sie den folgenden Aussagen zu?**

|  | Ich stimme voll zu |  |  |  | Ich stimme gar nicht zu |
| --- | --- | --- | --- | --- | --- |
| Die Online-Klausur war für mich stressiger als bisherige  Papier-basierte Prüfungen. |  |  |  |  |  |
| Ich schreibe lieber papierbasierte Prüfungen als Online-Klausuren. |  |  |  |  |  |
| Die Online-Klausur hat in mir ein gleich hohes Stressniveau  ausgelöst wie bisherige Papier-basierte Prüfungen. |  |  |  |  |  |
| Ich denke Online-Klausuren sollten eine wichtigere Rolle an der Fakultät für Medizin spielen. |  |  |  |  |  |
| In der Online-Klausur hatte ich Schwierigkeiten mich auf die  Fragen zu konzentrieren. |  |  |  |  |  |
| Ich bevorzuge Online-Klausuren, weil ich es gewohnt bin, am PC zu arbeiten. |  |  |  |  |  |
| Bei der Online-Klausur hat mir die soziale Interaktion mit meinen KommilitonInnen gefehlt. |  |  |  |  |  |

**Inwiefern stimmen Sie den folgenden Aussagen zu?**

|  | Ich stimme voll zu |  |  |  | Ich stimme gar nicht zu |
| --- | --- | --- | --- | --- | --- |
| Die Online-Klausur ist ein geeignetes Prüfungsformat für die Vorlesungsreihe Medizin des Alterns. |  |  |  |  |  |
| Die Online-Klausur hat nicht nur mein Fachwissen, sondern auch meine IT-Kompetenz getestet. |  |  |  |  |  |
| Die Online-Klausur ist eine zeitgemäße Prüfungsform. |  |  |  |  |  |
| Für die Online-Klausur war ein zu knappes Zeitlimit angesetzt. |  |  |  |  |  |
| Das Anforderungsniveau der Klausurfragen war durch die  Alternative der Online-Klausur unangebracht höher als in herkömmlichen Papier-basierten Prüfungen. |  |  |  |  |  |

**Inwiefern stimmen Sie den folgenden Aussagen zu?**

|  | Ich stimme voll zu |  |  |  | Ich stimme gar nicht zu |
| --- | --- | --- | --- | --- | --- |
| Ich bewerte es positiv, dass die Online-Klausur weniger Papier verbraucht. |  |  |  |  |  |
| Ich befürchte, dass mein Abschneiden in der  Online-Klausur durch eine schlechte Internetverbindung beeinträchtigt wurde. |  |  |  |  |  |
| Rückwirkend betrachte verfüge ich nicht über die notwendige  Ausstattung eine Online-Klausur zu schreiben. |  |  |  |  |  |
| Rückwirkend betrachtet habe ich außerhalb des Campus keine  angebrachte Umgebung, um eine Online-Klausur zu schreiben. |  |  |  |  |  |
| Die Online-Klausur war für mich leichter zugänglich als die Papier-basierte Prüfung. |  |  |  |  |  |
| Das verschiedene Fächer unterschiedliche Plattformen  (name oft he platform, EvaExam) zur Ausrichtung der Online-Klausur genutzt haben, hat zu meiner Belastung als Studierender beigetragen. |  |  |  |  |  |

**Inwiefern stimmen Sie den folgenden Aussagen zu?**

|  | Ich stimme voll zu |  |  |  | Ich stimme gar nicht zu |
| --- | --- | --- | --- | --- | --- |
| Technische Störungen der Prüfungsplattform haben den Klausurablauf beeinträchtigt. |  |  |  |  |  |
| Die Online-MC-Fragen waren zuverlässiger als Papier-basierte Prüfungen, da Übertragungsfehler vermieden wurden. |  |  |  |  |  |
| Papier-basierte Prüfungen sind fairer als die Online-Klausur. |  |  |  |  |  |

**Inwiefern stimmen Sie den folgenden Aussagen zu?**

|  | Ich stimme voll zu |  |  |  | Ich stimme gar nicht zu |
| --- | --- | --- | --- | --- | --- |
| Die Online-Klausur war genauso sicher, wie die Papier-basierte Prüfung. |  |  |  |  |  |
| Ich habe Vertrauen in die Datensicherheit der  Prüfungssoftware (name of the platform). |  |  |  |  |  |
| Es war in der Online-Klausur leichter zu betrügen als in  bisherigen Papier-basierten Prüfungen. |  |  |  |  |  |
| Hacker-Angriffe stellen ein akutes und ernstzunehmendes  Problem für (name oft he paltform) dar. |  |  |  |  |  |
| Personalisierter Link und Matrikelnummer haben ein  ausreichendes Sicherheitsmaß für die Online-Klausur geboten. |  |  |  |  |  |

**Inwiefern stimmen Sie den folgenden Aussagen zu?**

|  | Ich stimme voll zu |  |  |  | Ich stimme gar nicht zu |
| --- | --- | --- | --- | --- | --- |
| Die Online-Klausur konnte meinen Lernprozess unterstützen. |  |  |  |  |  |
| Die Online-Klausur war nur eine Spielerei, die mich nicht beim Lernen unterstützte. |  |  |  |  |  |
| Die Online-Klausur ging Hand in Hand mit Online-Learning. |  |  |  |  |  |
